# Comparative analysis of the influence of a high‐fat/high‐carbohydrate diet on the level of anxiety and neuromotor and cognitive functions in Wistar and DAT‐KO rats

**DOI:** 10.14814/phy2.13987

**Published:** 2019-02-19

**Authors:** Sergey A. Apryatin, Vladimir A. Shipelin, Nikita V. Trusov, Kristina V. Mzhelskaya, Victoria S. Evstratova, Natalya V. Kirbaeva, Jorge S. Soto, Zoia S. Fesenko, Raul R. Gainetdinov, Ivan V. Gmoshinski

**Affiliations:** ^1^ Federal Research Centre of Nutrition and Biotechnology Moscow Russia; ^2^ Institute of Translational Biomedicine St. Petersburg State University Petersburg Russia

**Keywords:** Behavioral reactions, dopamine, knockout models, neuromotor function, rats

## Abstract

We compared anxiety, neuromotor, and cognitive functions in mutant rats with different allelic variants of dopamine transporter DAT knockout receiving balanced or excess in fat and fructose diet. The experiments were performed in DAT−/− homozygotes, DAT+/− heterozygotes, and DAT+/+ wild type rats. The genotype of DAT‐KO rats was confirmed by restriction analysis of DAT gene compared to behavioral responses in the open field test (OF). Animals in the first groups of each strain were fed a balanced AIN93M diet; and those in the second groups with a high‐fat/high‐fructose diet. Neuromotor function was studied as grip strength, and behavioral responses were assessed in the elevated plus maze and conditioned passive avoidance response tests. The mass of the internal organs and white and brown fat, as well as selected lipid and nitrogen metabolism parameters in blood plasma were determined at the end of the experiment. DAT−/− had the highest specific grip strength, and showed an increase in initial exploratory activity in comparison with DAT+/− and DAT +/+. The exploratory activity was significantly reduced in the second test compared to the first one in DAT−/− and DAT+/− of first but not second group. Anxiety decreased with age in the second groups of DAT+/− and DAT+/+ (but not in DAT−/−) and was higher in DAT+/+ than in DAT+/− and DAT−/−. Excess fat and fructose resulted in the deterioration of short‐term memory in DAT+/+. Lipidomic indices of blood plasma were less responsive to diet in DAT−/− and DAT−/+ in comparison to DAT+/+. The increased AsAT/AlAT activity ratio in DAT−/− compared with those in DAT+/+ suggests the activation of catabolism activity in the mutants. The consumption of excess fat and fructose significantly modified the effects produced by DAT gene allelic variants presumably due to the influence on the processes of dopamine metabolism.

## Introduction

Dopamine and the dopaminergic system of the forebrain play a decisive role in the formation of satiety and food reward underlying food intake and eating behaviors (Auriemma et al. 2018; Manning et al. 2018; Ramos‐Lopez et al. 2018). According to a number of studies, the consumption of high‐fat and high‐carbohydrate diets, predisposing to obesity, causes persistent changes in the metabolism of dopamine, leading to disruption of the formation of a sense of food saturation (Lee et al. 2010; Meyers et al. 2017; Robertson and Rasmussen 2017). In this regard, dopaminergic mechanisms of food‐related behavior are considered as promising targets for pharmacological interventions in the treatment of obesity, complementary to traditional dietary therapy, and lifestyle correction (Bessesen and Van Gaal 2018). The use of animal lines with alterations in genes critical for the exchange of dopamine is a convenient way of assessing the roles of the dopaminergic system reflected in behavioral responses to food imbalances. One such line is the DAT‐KO rat strain, representing a knockout of the gene encoding the plasma membrane transporter of dopamine, DAT, which is responsible for its clearance from synapses of neurons (Efimova et al. 2016; Cinque et al. 2018). Due to this genetic defect, dopamine is not utilized and progressively accumulates in the synaptic cleft. In these animals, this leads to the abolition of the suppression of motor and exploratory reactions. DAT‐KO rats exhibit specific behavioral characteristics such as sharply increased motor activity and long‐lasting motor excitation, which manifests as numerous repeated motor reactions (head turns, flexion and extension of limbs, etc.) (Adinolfi et al. 2018; Leo et al. 2018). These manifestations are in contrast with known behavioral shifts, such as depression, characteristic of the development of alimentary obesity (Bieliński et al. 2017; Winther et al. 2018; Caldwell and Sayer 2018). Therefore, DAT‐KO animals (both those homozygous and heterozygous for the defective gene) are an attractive model for studying the effects of a hypercaloric diet on food behavior mediated by dopaminergic neurons.

Behavioral reactions are among the most informative indicators that show the development of obesity in the laboratory in vivo models of alimentary‐dependent diseases. The consumption of high‐fat and high‐carbohydrate diets leads to an increase in the mass of fat tissue and the development of inflammatory reactions with shifts in the production of messengers (such as leptin, ghrelin, adiponectin, and pro‐ and anti‐inflammatory cytokines) (Simonson 2001; Vieira et al. 2009; Rask‐Madsen and Kahn 2012). All these changes can influence the activity of the serotonergic and dopaminergic neurons in the arcuate nucleus of the hypothalamus, which are associated with the neurons in the higher forebrain and are responsible for behavioral reactions (including food search activity and anxiety level), as well as the states of short‐term and long‐term memory (Kruger et al. 2012; Vucetic et al. 2012; Burke and Heisler 2015).

The purpose of this study was to compare the level of anxiety and neuromotor and cognitive function of DAT mutant and wild‐type rats with obesity induced by consumption of a diet with an increased proportion of fats and fructose.

## Materials and Methods

The studies were carried out on male rats aged 10–12 weeks. DAT‐KO knockout line (DAT −/− homozygotes and DAT +/− heterozygotes) rats were obtained from the vivarium at the Institute of Translational Biomedicine of St. Petersburg State University. An outbred line of DAT +/+ rats was purchased from the “Stolbovaya” Nursery of Laboratory Animals at the Scientific Center for Biomedical Technology of the Federal Medical and Biological Agency. Wistar rats are the parental line used to develop DAT‐KO rats and are the most similar to them with regard to allelic gene composition, except *DAT* so named “wild‐type” rats.

The experimental design was approved by the Animal Ethics Committee of the Federal Research Centre of Nutrition and Biotechnology (Protocol No. 04 from 20/04/2017) in accordance with the order of the Ministry of Health and Social Development of the Russian Federation dated 04/01/2016, N199 “On Approval of the Rules of Laboratory Practice.”

Phenotyping of DAT‐KO homozygotes and heterozygotes for the knockout *DAT* gene was performed based on their behavioral responses in the open field (OF) test. The studies were carried out on Panlab Harvard Apparatus equipment (Spain). Repeated testing of the animals in the OF test was performed on the 52nd day after the beginning of feeding with the experimental diets. The results of phenotyping were verified after the removal of animals from the experiment by means of genotyping by *DAT* gene DNA amplification with PCR followed by restriction enzyme BtsIMutI digestion and electrophoretic separation. The details of the method are described in Leo et al. (2018). After the pretyping, the animals were divided into six groups. Animals in the first (DAT −/−, *n* = 4), third (DAT +/−, *n* = 12), and fifth (DAT +/+, *n* = 8) groups received a control diet based on AIN93M with the following changes: (1) there were excluded tert‐butylhydroquinone and sucrose; (2) cornstarch amounted to 60% instead 62.5%, casein amounted to 20% instead 14%, sunflower oil with lard 10% was used instead of soybean oil; (3) sodium metasilicate amounted to 0.63 g/kg in the salt mixture instead 1.45 g/kg; (4) the diet was supplied with vitamin E without taking into account its content in soybean oil. The rats in the second (DAT −/−, *n* = 6), fourth (DAT +/−, *n* = 9), and sixth (DAT +/+, *n* = 8) groups received a high‐fat‐carbohydrate diet (HFCD) with 30% fats by dry weight by replacing part of the starch and a 20% fructose solution instead of drinking water. Both diets were supplied ad libitum. The assessment of muscle tone state of the rats was carried out on the 25th day by determining the grip strength of the front paws. The grip strength was determined in mN by measuring the maximum dynamometer readings over two repetitions (at the moment when the animal is uncoupled from the frame).

Indices of short‐term and long‐term memory of the animals were studied in the conditional reflex of passive avoidance (CRPA) test on the 37th, 38th, and 59th days by using equipment from Panlab Harvard Apparatus. During the training, the animals were initially placed in a lighted compartment of the apparatus with their backs to the dark compartment. Under the influence of exploratory behavior and a natural preference for dark areas of space (photophobia), the rats quickly entered the dark compartment. The latent period (LP) of staying in the lighted compartment of the chamber was recorded. As soon as the animal passed into the dark compartment of the chamber, it received electrocutaneous irritation on the paws (not more than 8 sec, current 0.3 mA, resistance 150 ohms). Then, the rat was immediately moved to a separate cage until all animals from the cage were tested. Twenty‐four hours after the training, the animals were retested to fix the memory trace in the same chamber, but without a current supply. The rat was placed in the lighted compartment of the chamber in the starting position, and the LP was determined in the bright compartment before the rat went into the dark one. If the animal did not go into the dark compartment of the chamber for 180 sec, it was believed that the memory trace was fixed. The retention of the memory trace was tested after 3 weeks using the same procedure.

Animal anxiety levels were assessed by using the elevated plus maze (EPM) test. The test allows the assessment of the degree of the emotional reaction of fear and anxiety, motor activity (mobility), and the rate of orienting reactions. By using the Smart 3.0.04 video surveillance system, the following data were recorded: the time spent in the open arms (OAs) and closed arms (CAs) of the maze by the animal; the latency time of the entrance to the CAs; the number of transitions between the zones; the path (distance) traversed in the maze. The time the laboratory animals spent in the maze was 300 sec. Testing was conducted before the beginning of feeding (Test 0) and on the 36th (Test I) and 57th days (Test II) after the beginning of the feeding.

On the 62nd day, animals were decapitated under ether anesthesia. The mass of organs, retroperitoneal and brown fat tissue was determined on laboratory scales with an accuracy of ±0.01 g; the relative mass of internal organs was calculated as a % of body weight. The blood was collected in tubes with a 1.0% heparin solution in 0.15 mol/L NaCl (1:10 by volume). The plasma was separated by centrifugation, and biochemical indices (glucose, triglycerides, cholesterol, etc.) were examined on a Konelab 20i biochemical analyzer (Finland) using standard techniques such as kinetic methods recommended by the International Federation of Clinical Chemistry and Laboratory Medicine for AlAT, AsAT and lipase activities, glucose oxidase test for glucose, Trinder reaction with glycerol‐3‐phosphate oxidase for triglycerides, UV kinetic urease method for urea, enzymatic endpoint (CHOD) method for cholesterol, direct endpoint method without immunoinhibition for lipoproteins.

Statistical processing of data was carried out using a three‐way ANOVA test, two‐tailed Fisher's angular transformation criteria (for alternative indices), and the nonparametric rank criteria of the Wilcoxon–Mann–Whitney test for pairwise comparisons. The calculations were performed using the statistical package of SPSS®16.0 and Excel for Windows. Significance was assessed at the level of *P* < 0.05.

## Results

### Phenotyping of the animals in the open field test

**Figure 1:**
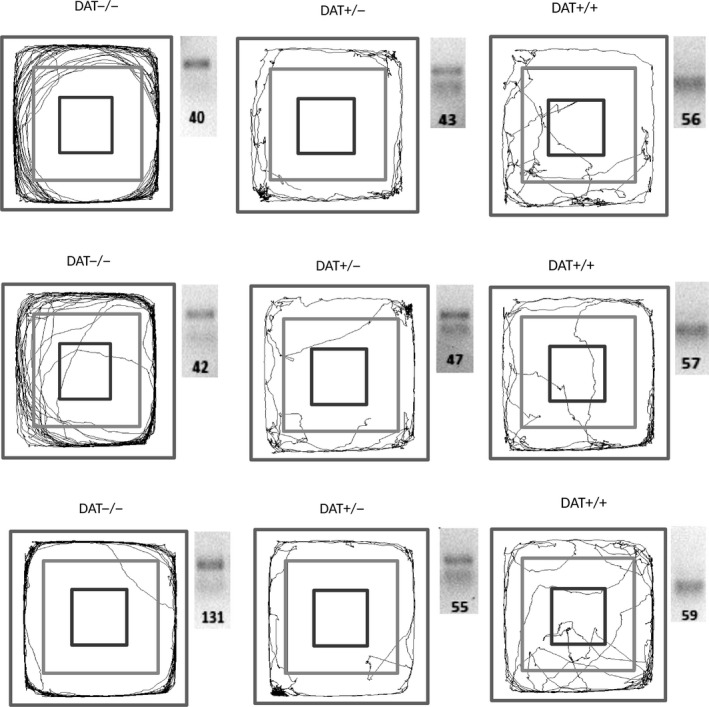
Representative trajectories of the DAT‐KO (DAT −/− and DAT +/−) and DAT +/+ rats in the OF test during 5 min of observation and the results of amplified DAT gene restriction analysis in these animals.

Figure 1 shows an example of the revealing of rats’ genotypes depending on the trajectories of their movement in the OF test during 5 min of observation. It can be seen that the DAT −/− rats were characterized by markedly increased mobility, which was manifested in a unidirectional (mainly counter‐clockwise) rapid movement within zone 4 (periphery) of the arena. A similar pattern was less pronounced for the DAT +/− heterozygotes that was manifested in a smaller traveled distance with the presence of some stops (delays) in the corners of the arena accompanied by standing and acts of grooming. The DAT +/+ rats were characterized by an unsystematic multidirectional movement within the field. In addition to staying within zone 4, the rats made numerous visits to zones 3 and 2. At the same time, the number of delays in movement and racks on rear limbs were less than those in the DAT +/− group. Genotyping demonstrated in DAT−/− rats one band of higher molecular weight for knocked out *DAT*, in DAT+/− rats − one band of lower molecular mass for wild‐type (BtsIMutI digestible) *DAT,* and in DAT +/− rats − both types of these bands (Fig. 1).

**Figure 2:**
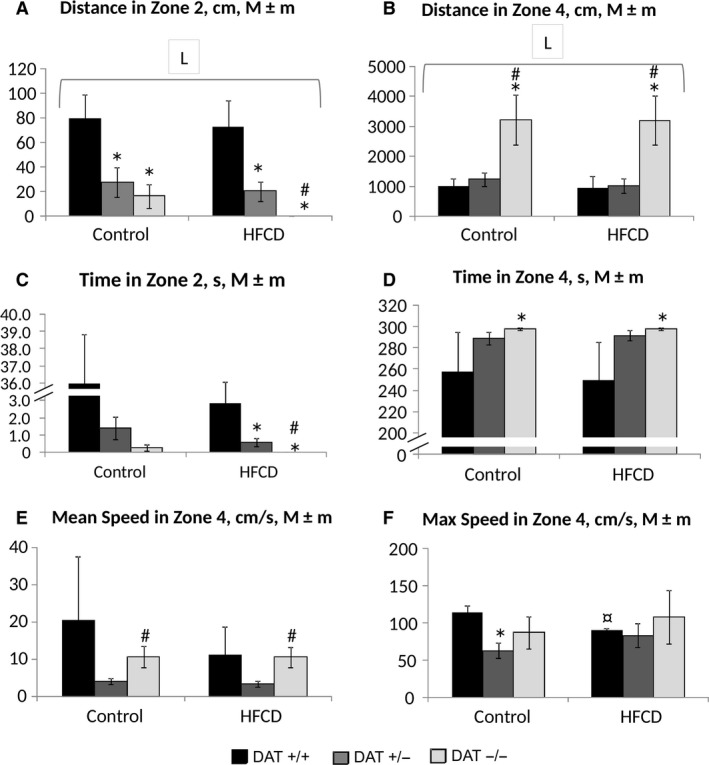
Results of the rats’ testing in the OF on the 52nd day of the experiment. (A) the distance traveled in zone 2 (center of the field), cm; (B) the distance traveled in zone 4 (the periphery of the field), cm; (C) the time spent in zone 2, sec; (D) the time spent in zone 4, sec; (E) the average speed in zone 4, cm/sec; (F) the maximum speed in zone 4, cm/sec. The abscissa axis – the group of animals, the ordinate axis – M ± m index in the appropriate units. The number of animals per group ‐ see Table 1. *The difference compared with the data from DAT −/− rats is significant; ^#^The difference compared with the data from DAT +/− rats is significant, ^¤^The difference compared with the corresponding control group is significant, *P* < 0.05, the Wilcoxon–Mann–Whitney test; horizontal bracket – ANOVA *P* < 0.05 for “genotype” (L, line) by the covered range of values.

The data in Figure 2 show that the patterns observed in the first trial in the OF test were essentially the same when the test was repeated. The DAT −/− rats had the highest traveled distance in zone 4 (periphery of the site) and the total traveled distance in comparison with both DAT +/− and DAT +/+ rats (*P* < 0.001 for “genotype”; three‐way ANOVA test). In contrast, DAT +/+ rats spent more time in the center of the field (zone 2) and less time in zone 4 than the knockout rats (*P* < 0.001 for “genotype”). DAT +/− rats were characterized by the lowest mean and maximum speed (i.e., apparently, their movement was more uniform over time). There were no significant differences in the OF test when comparing animals that received or did not receive the HFCD (ANOVA *P* > 0.1 for “diet”). The exception was a significant decrease in the maximum speed at the periphery of the field in DAT +/+ rats. The DAT−/− rats receiving HFCD showed significantly less time spent at the center of the site, zone 2 compared with DAT +/− and DAT +/+ animals.

### Examination of neuromotor function

Table 1 presents the results of the determination of muscle compression force (the grip strength of the front paws) per unit body weight (b.w.). The muscle compression force is one of the informative characteristics of a neuromotor function of animals. Changes in this indicator can have both a central nature and indicate a violation of the energy metabolism processes in muscle tissue (Coradinia et al. 2015; Fujiwara et al. 2017; Martinez‐Huenchullan et al. 2018). As follows from the data obtained, DAT −/− rats were characterized by a higher specific muscular compression force (per unit b.w.) compared with both DAT +/− and DAT +/+ rats, whereas there was no impact of the HFCD on this indicator (*P* < 0.001 for “genotype”; *P* > 0.1 for “diet”; three‐way ANOVA test).

**Table 1:** Results of the evaluation of neuromotor function of rats at day 25 of the experiment

Rat group	Diet	Number of rats	Muscle compression force, N/kg of b.w. (M±m, median, interval of changes)^1^
DAT −/−	Control	4	43.2 ± 5.6 44.4 (28.6–55.3)
DAT −/−	HFCR	6	49.3 ± 1.0 48.9 (33.8–54.0)
DAT +/−	Control	12	31.6 ± 1.8 33.9 (17.5–39.3)
DAT +/−	HFCR	9	29.9 ± 2.1^2^ 30.4 (19.3–38.5)
DAT +/+	Control	8	31.3 ± 2.3 31.4 (22.4–41.9)
DAT +/+	HFCR	8	30.4 ± 2.5^2^ 30.6 (22.1–40.7)

1 Three‐way ANOVA *P* < 0.001 for «genotype».

2 *P* < 0.05, Mann–Whitney test when compared with DAT −/− rats on the corresponding diet.

### Assessment of anxiety level in the EPM test

**Figure 3:**
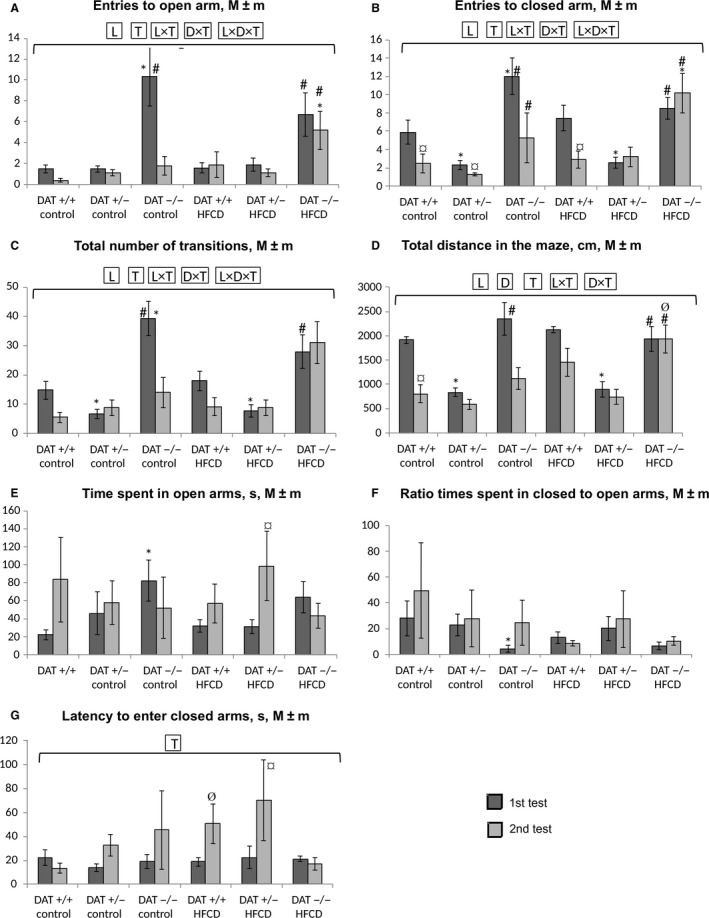
Results of testing the level of anxiety, search activity, and mobility of rats in the EPM test in the first (on the 7th day) and second (on the 50th day) testing. (A) The number of OA visits; (B) the number of CA visits; (C) the total number of transitions between the maze zones; (D) the total distance traveled in the maze, cm; (E) the time spent in OAs, seconds; (F) the ratio of the CA/OA time; (G) the latency time before the first entry into the CA. Abscissa axis – groups of animals; Ordinate axis – the mean ± standard error of the indicator in the appropriate units. The number of animals per group – see Table 1. *The difference compared with the data from DAT −/− rats is significant; ^#^The difference compared with the data from DAT +/− rats is significant; ^¤^The difference compared with the data from first test in corresponding group is significant; ^Ø^The difference with the animals receiving the control diet is significant, *P* < 0.05 (the Wilcoxon–Mann–Whitney test); horizontal bracket – ANOVA *P* < 0.05 for “genotype” (L, line), “diet” (D), test (T) and their combinations by the covered range of values.

As follows from the data presented in Figure 3, the DAT −/− rats were characterized by an initial increase in exploratory activity in comparison with the DAT +/− rats. This behavior was manifested as a significantly greater number of visits to the OAs (Fig. 3A) and CAs (Fig. 3B) of the maze, and the total number of transitions between zones (Fig. 3C) during the first test (ANOVA *P* < 0.05 for “genotype”). During repeated testing, the differences in the number of visits to the CAs and the total number of transitions were maintained between the DAT −/− and DAT +/− rats. Compared to the first test, the exploratory activity was significantly reduced in the second test (Fig. 3B) in all DAT +/+ and DAT +/− receiving HFCD, but not in any DAT −/− rats. It is noteworthy that the direction of change in mobility at the second test in comparison with the first one was opposite for DAT −/− (Fig 3B and C) and DAT +/− (Fig. 3B), but not for DAT +/+ receiving different rations (ANOVA *P* < 0.05 for “genotype × test” and “genotype × diet × test”). As a result, the differences in search activity noticed between DAT−/− and DAT +/− at the first test were leveled at the second one.

The overall mobility of the rats was determined by the total distance traveled in the maze (Fig. 3D). This index was significantly reduced in the second test in comparison with the first in the DAT +/+ rats but not the DAT +/− and DAT−/− rats (*P* < 0.05 for “genotype”, “test,” and “genotype*test,” three‐way ANOVA). In this case, the DAT +/− rats were characterized by the lowest total mobility in the first test in comparison with both the DAT −/− and DAT +/+ rats. The consumption of the HFCD caused a increase in mobility at the second test ANOVA (*P* < 0.05 for “diet*test”) most noticeable in the DAT −/− rats.

The anxiety level of the animals was estimated by how long the rats stayed in the CAs (Fig. 3E) and OAs of the maze, by the ratio of these indices (Fig. 3F), and the latency time to the first entrance into the CAs (Fig. 3G). It can be seen (Fig. 2E) that when the control diet was consumed, anxiety decreased in DAT +/+ rats in the second test compared with the first one at the level of the trend (*P* < 0.1), while in the DAT −/+ and DAT −/− rats, this finding was not observed. In contrast, in the HFCD‐consuming DAT +/− rats (Fig. 3E) and DAT+/+ (Fig. 3G), but not in DAT −/− rats, anxiety decreased in the second test compared to the first one. Anxiety in the DAT −/− rats was significantly lower in the first test in comparison with the DAT +/+ rats consuming the control diet (Fig. 3E,F). In the DAT +/−, but not in DAT −/− rats receiving the HFCD, anxiety in the second test significantly lowered in comparison with the first one (Fig. 3G). Prolonged HFCD consumption made anxiety lower in comparison with the control group only in DAT+/+ rats (Fig. 3G).

Thus, it can be concluded that the level of anxiety, as determined by key indicators in the EPM test, was higher in the DAT +/+ rats than in the DAT +/− and DAT −/− rats. Against the background of HFCD consumption, DAT +/− rats were less affected in comparison with DAT −/− rats.

### Determination of indicators of short‐term and long‐term memory in the CRPA test

As follows from the data presented in Table 2 during the first test (development of the CRPA after electrocutaneous stimulation of the paws after entering the dark compartment), there was a significantly lower (*P* < 0.05) LP in the DAT −/− rats compared with both DAT +/− and DAT +/+ rats. This finding was consistent with the increase in the level of search activity in the DAT −/− rats identified in the EPM test. The consumption of HFCD by animals led to a decrease in the severity of this effect (differences between groups become statistically nonsignificant). The second testing after the first day was an indicator of short‐term memory and the degree of CRPA fixation. In this test, the number of animals that did not enter the dark compartment was significantly lower in the DAT +/+ rats than in the DAT +/− rats under the HFCD condition but not under the control diet condition. The data obtained allow the conclusion that the consumption of HFCD together with the decrease in anxiety level shown in the EPM test results in the deterioration of short‐term memory in DAT +/+ rats but not DAT +/− rats. The degree of CRPA fixation in animals (excluding DAT +/− rats) in the second test did not exceed 50%. Consequently, in carrying out the third test (long‐term fixation of the memorable trace), the number of rats was insufficient to conduct statistical analysis of the data.

### Integral and biochemical indicators of animals

**Figure 4:**
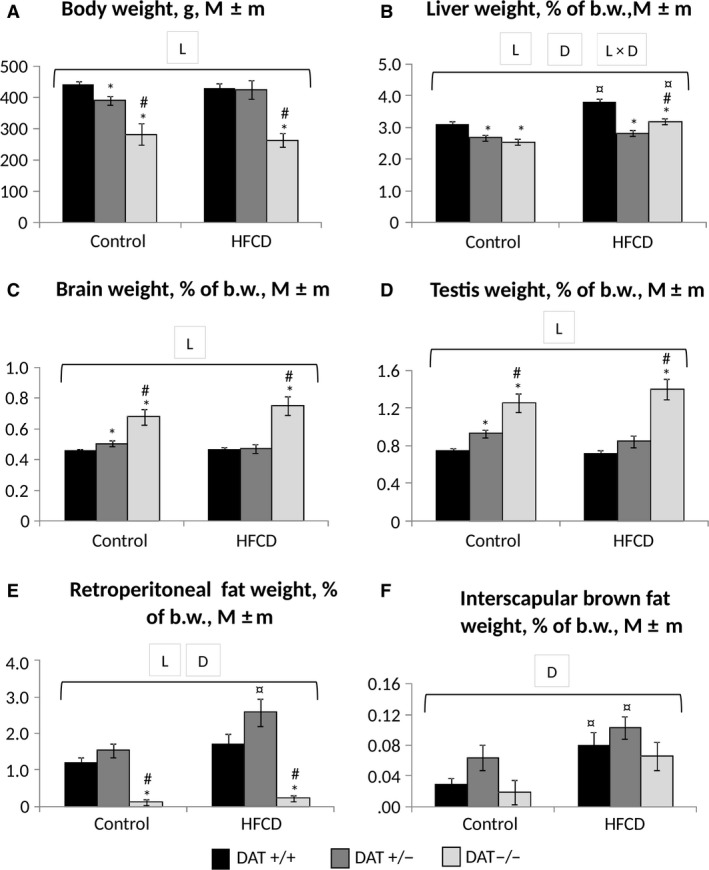
Integral indices of rats at the end of the experiment. (A) Body weight, g; (B–F) relative weights of the liver, brain, gonads, white and brown adipose tissue, % of body weight. Abscissa axis – groups of animals; ordinate axis – the mean ± standard error of the indicator in the appropriate units. The number of animals per group – see Table 1. *The difference compared with the data for DAT −/− rats is significant; ^#^The difference compared with the data for DAT +/− rats is significant; ^¤^The difference compared with the animals receiving the control diet is significant, *P* < 0.05 (Wilcoxon–Mann–Whitney test); horizontal bracket – ANOVA *P* < 0.05 for “genotype” (L, line), “diet” (D) and their combination by the covered range of values.

Figure 4 shows the data of body and internal weight at the end of the experiment. As seen, the DAT ‐/‐ rats consuming both experimental diets significantly lagged behind in their final b.w. compared with both DAT +/− and DAT +/+ rats (*P* < 0.001 for “genotype,” ANOVA). At the same time, DAT −/− rats were characterized by a significantly reduced relative weight of the liver and increased weight of the brain and gonads in comparison to the rats of the other examined lines (*P* < 0.001 for “genotype,” ANOVA). The consumption of HFCD resulted in a significant (*P* < 0.05) increase in the weight of the liver in DAT −/− and DAT +/+ rats in comparison to the corresponding control groups. Both diet and animals’ line influenced the liver weight (*P* < 0.05 for “genotype * diet”, ANOVA). In DAT −/− rats treated with HFCD, there was a significant increase in cardiac mass (24%) compared with DAT +/+ rats and kidney (21%) compared to DAT +/− rats (data are not presented). Both genotype and diet strongly influenced the heart weight (ANOVA *P* < 0.05 for “genotype” and “diet”). DAT −/− rats from a control group showed a significant decrease in the accumulation of retroperitoneal fat stores in comparison with the rats of the other two lines (*P* < 0.001 for “genotype,” ANOVA). HFCD consumption resulted in retroperitoneal fat accumulation which was most pronounced in DAT +/− and DAT +/+ (*P* < 0.001 for “diet,” ANOVA). In contrast to this, interscapular brown fat stores were strongly influenced by diet but not animal's line (*P* < 0.001 for “diet”; *P* > 0.1 for “genotype,” ANOVA).

**Figure 5:**
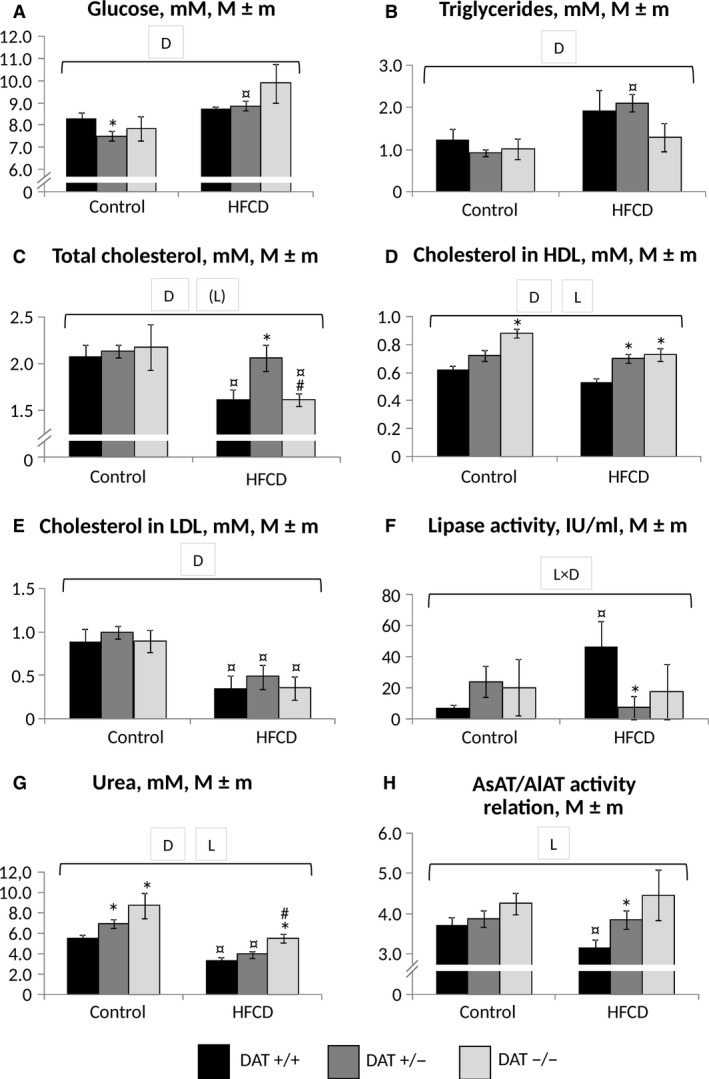
Biochemical indices of blood plasma in rats at the end of the experiment. (A) Glucose; (B) triglycerides; (C) total cholesterol; (D) HDL cholesterol; (E) LDL cholesterol; (F) lipase (total lipolytic activity); (G) urea; (H) AsAT/AlAT activity ratio. Abscissa axis – groups of animals; ordinate axis – the mean±standard error of the indicator: concentration, mmol/L (A–E, G), activity (F), dimensionless ratio (H). The number of animals per group – see Table 1. *The difference compared with the data for DAT −/− rats is significant; ^#^The difference compared with the data for DAT +/− rats is significant; ^¤^The difference compared with the animals receiving the control diet is significant, *P* < 0.05 (Wilcoxon–Mann–Whitney test) horizontal bracket – ANOVA *P* < 0.05 for “genotype” (L, line), “diet” (D) and their combination by the covered range of values; (L)‐ *P* < 0.1.

As follows from the data of blood plasma biochemical parameter results (Fig. 5), the intake of HFCD resulted in an increase in the blood glucose level (Fig. 5A) in both DAT −/+ and DAT −/− rats (the difference in the latter case was statistically insignificant due to the small number of animals in the group). However, in DAT +/+ rats, the hyperglycemic effect of HFCD was significantly less pronounced. In general, diet but not strain significantly influenced glucose level (*P* > 0.1 for “genotype”; *P* < 0.001 for “diet” ANOVA). In all three rat strains, the intake of HFCD was accompanied by an increase in triglyceride (Fig. 5B) levels (*P* < 0.005 for “diet,” ANOVA).

**Table 2:** Results of CRPA test in rats

Group	Line/genotype of rats, diet	First test		Second test (after 24 h)	
		Time to entry, sec, M ± m	Number of not logged in/total number (part, %)	LP, sec, M ± m	Number of not logged in/total number (part, %)
1	DAT +/+, control	10.5 ± 1.7	0/8 (0)	112 ± 28	4/8 (50)
2	DAT +/+, HFCR	10.4 ± 2.0	0/7 (0)	46.9 ± 23.4	1/7 (14)
3	DAT +/−− control	22.0 ± 14.5^2^	1/12 (8)	120 ± 25	7/11 (65)
4	DAT +/−− HFCR	40.0 ± 18.4	1/9 (11)	119 ± 30	5/8 (62)^3^
5	DAT −−/−− control	2.05 ± 0.51^1^	0/4 (0)	93.7 ± 49.8	2/4 (50)
6	DAT −−/−− HFCR	7.47 ± 2.30	0/6 (0)	65.5 ± 36.4	2/6 (33)

1 The difference compared with DAT +/+ rats, control (group 1) is significant, *P* < 0.05.

2 The difference compared with DAT −/− rats, control (group 5) is significant, *P* < 0.05.

3 The difference compared with DAT +/+ rats, HDR (group 2) is significant, *P* < 0.05.

The content of total cholesterol (Fig 5C) in the blood plasma significantly responded to the consumption of HFCD in DAT +/+ rats, in whom a decrease in total cholesterol was observed. HDL cholesterol (Fig. 5D) content in these rats consuming HFCD was the lowest in comparison with the animals of both knockout lines. ANOVA analysis demonstrated that both line (*P* < 0.05) and diet (*P* < 0.001) influenced this index. The calculated content of LDL cholesterol (Fig. 5E) decreased with the use of HFCD in all three animal lines; the decrease was significant in the DAT +/− and DAT +/+ rats (*P* < 0.001 for “diet,” ANOVA). Attention is drawn to the sharp decrease in lipolytic activity (Fig. 5F), which is present in heparin plasma, mainly by hepatic and lipoprotein‐lipase (Eckel et al. 1988), in DAT +/− rats to almost undetectably low values, in contrast to DAT +/+ rats, in which it was increased (*P* < 0.02 for “genotype * diet,” ANOVA). A significantly decreased urea level (Fig. 5G) was noted in DAT +/+ rats in comparison with DAT−/− and besides this in all groups of rats treated with HFCD (*P* < 0.001 for “genotype” and “diet,” ANOVA).

An increase in the activity of the cardiac and liver transaminases AlAT and AsAT is an indicator of the heart and liver cell damage. Accordingly, AsAT and AlAT alone did not respond to the intake of HFCD in all groups (*P* > 0.1, ANOVA). However, AsAT activity was significantly increased (*P* < 0.001 for “genotype,” ANOVA) in DAT −/− rats compared with DAT +/− and DAT +/+ rats by 28% and 38% in controls and by 23% and 58% in HFCD consumption groups, respectively (data not shown). At the same time, the dimensionless ratio (Fig. 5H) of AsAT/AlAT activity (so named DeRitis ratio, considered a biomarker of the ratio of the transamination processes in the liver and muscles)(Botros and Sikaris 2013) was significantly (*P* < 0.05) reduced in DAT +/+ rats treated with HFCD compared to the same animals that received the control diet and the corresponding DAT +/− group. The difference in the activity ratio of AsAT/AlAT to DAT −/− rats was also statistically nonsignificant, apparently due to an insufficient number of these animals. The results of increased AsAT activity in DAT −/− rats is consistent with the decrease in body weight in comparison with the DAT +/− and DAT +/+ rats and with the signs of liver steatosis observed in DAT +/+, but not in DAT −/− and DAT +/−, as detected by light microscopy (data in a separate publication).

## Discussion

The results of the integral index analysis (body weight, relative weight of organs) revealed clear differences in rats with DAT −/− and DAT +/+ genotypes for body weight, liver, retroperitoneal and interscapular fat, brain and testis relative weight. Increased resistance of DAT gene knockout animals to the effect of excessive energy value factors of the diet is manifested in DAT −/− rats as reduced body weight, relative weight of liver, and white retroperitoneal fat in comparison with DAT +/+ rats both on the experimental diet and on control rations. This finding may indicate the genetically determined effect of excess (fivefold excess of the norm) amounts of dopamine in the brain of this knockout line of DAT −/− rats on the neuroregulatory function, which results in motor hyperactivity in these animals (Leo et al. 2018). Prolonged increased physical activity in DAT −/− and in less extent in DAT +/− rats can provoke different changes in metabolism by analogy with the way it was observed on the model of leptin‐deficient animals (McGee‐Lawrence et al. 2017).

The effect of HFCD on the main biochemical parameters of carbohydrate and lipid metabolism (glucose, triglycerides, total HDL and LDL cholesterol) was generally similar in both knockout lines and wild‐type rats. However, plasma lipolytic activity responded to HFCD in DAT −/− and DAT+/− in a way which was different from wild type rats. This may indicate the ability of the DAT knockout genotype to block changes in lipolytic activity caused by excess fat and carbohydrates, which predispose to the development of insulin resistance and metabolic syndrome (Kobayashi et al. 2007).

At the same time, the acceleration of the processes of nitrogen metabolism associated with the involvement of tissue proteins in the processes of catabolism leads to an elevated level of AsAT activity in blood plasma. According to the data (Pósa et al. 2015), the increase in AsAT can be considered not only as an indicator of damage to liver cells (accompanied by the release of this enzyme into the systemic circulation) but also as a marker for enhanced transamination of oxaloacetate with the formation of aspartic acid (aspartate). The latter is a transport molecule for the delivery of oxaloacetate in the mitochondria as an activator of catabolic processes in the tricarboxylic acid cycle. Ammonia, the product of deamination of amino acids, connects with a molecule of carbon dioxide with the participation of two ATP molecules to convert into carbamoyl phosphate and then into urea in the ornithine cycle. This finding is confirmed by increased levels of urea in plasma from DAT −/− rat in comparison with that of DAT +/+ rats, regardless of the consumed diet (Fig. 5).

A statistically significant increase in the levels of urea in plasma in DAT −/− rats compared to DAT +/+ rats can also be explained by the activation of carbohydrate and alanine catabolism in the glucose‐alanine shunt in genetically mediated elevated energy losses in these animals. At the same time, the consumption of HFCD reduced the level of blood urea consistently in all three rat lines, which may be a manifestation of the suppression of protein catabolism and the ornithine‐citrulline cycle. This is in consistency with changes in urea levels which were observed in rats with obesity, caused by consumption of a “cafeteria diet” similar to the HFCD (de Castro Ghizoni et al. 2013).

Simultaneously the data obtained in the present study may mean that DAT−/− are unable to develop a metabolic block in the ornithine‐citrulline cycle, observed in wild‐type rats when consuming a diet of high‐energy density (Sabater et al. 2014).

The most noticeable increase in the DeRitis ratio (AsAT/AlAT) in homozygous and heterozygous DAT‐KO rats compared to wild‐type rats (DAT +/+) against the background of HFCD consumption indicates an increase in the catabolism of amino acids in skeletal muscles in relation to the liver under conditions of increased motor activity and the associated energy costs, caused by the genetic defect in dopamine clearance. According to a number of studies, the DeRitis ratio increases in the condition of elevated energy expenditure caused by physical exercise (Martinez‐Huenchullan et al. 2018) and, on the contrary, decreases with the development of obesity and metabolic syndrome (Nikniaz et al. 2018). At the same time, a decrease in this ratio is considered as a marker of the risk of liver steatosis in human and animals (Taylor et al. 2018; Yang et al. 2018).

Identified features in the metabolic reactions of DAT−/− rats are associated with a genetically determined dysfunctional clearance of dopamine from the synapses of these animals (Leo et al. 2018). The dopaminergic system plays an important role in the regulation of eating behavior and related metabolic changes. Dysfunction of the dopaminergic system was identified in obesity, both in animals and in humans. Long‐term consumption of high‐fat diets leads to a change in the expression of the key dopamine genes in the hypothalamus (Lee et al. 2010). Reduced expression and function of the dopamine D2 receptor within the mesocorticolimbic contour (Johnson and Kenny 2010), reduced the concentration of dopamine in the striatum in rats and humans with obesity (Geiger et al. 2009) and suppressed the expression of the D1 receptor in the nucleus accumbens in rats genetically susceptible to excess body weight (Alsio et al. 2010), indicate the role of reduced levels of DA transmission as a link in the vicious circle that is formed during the development of alimentary obesity. The present study disclosed increased intensity of catabolism processes and their reduced sensitivity to the action of the HFCD in DAT −/− knockout animals, that is in consistency with the existence of a central regulation contour for catabolic pathways mediated by dopaminergic neurons of the central nervous system.

The differences in behavioral responses determined by knockout of the DAT gene were most pronounced in animals in the open field test, which has been previously chosen as a criterion for the genotype pretyping of rats. The delayed clearance of dopamine in the DAT −/− homozygotes resulted in a suppression of inhibition of motor reactions, which resulted in a prolonged motor activation, manifested as rapid translational movements of the animals on the periphery of the field and a reduction in the number of stops and exits to the center (Cinque et al. 2018).

Studies of muscle tone showed a higher level of specific muscular compression force for DAT −/− knockout rats in comparison with both DAT +/− and DAT +/+ rats. These results were obtained independently of the diet used. This fact can be explained by the genetically mediated physiological effect of excessive amounts of dopamine and its metabolites (noradrenaline and epinephrine) in the brain of homozygous rats with the DAT −/− genotype on the activation of fatty acid biosynthesis in adipose tissue, the rate of glucose utilization (catabolism) in muscle and liver tissue (glucose‐alanine shunt) (Manning et al. 2018; Rospond et al. 2018), and as a result, an increase in the degree of skeletal muscle contraction, including an increase in muscle contraction force.

The results of the first EPM test showed that rats with the DAT −/− genotype initially showed an increase in exploratory activity compared to the DAT +/− rats that manifested as a significant increase in the number of visits to the OAs (Fig. 3A) and CAs (Fig. 3B) of the maze and the total number of transitions between the zones (Fig. 2C). Such could be the reason for the genetically mediated high level of dopamine in the brain of young DAT −/− rats (Cinque et al. 2018). With age, with a control diet, the exploratory activity of rats DAT −/− decreased. The exploratory activity in DAT +/+ rats also decreased with age but during the first test was higher than that in DAT +/− but lower than that in DAT −/− rats. Consequently, even against a background of genetic differences between DAT +/+ and DAT −/− rats, exploratory activity differences were leveled with age, which can mean modulation of transcription activity of some genes determining neuroregulatory processes and food‐related behavior. This assumption needs confirmation in genomic, transcriptomic, and proteomic studies of the liver and brain of animals of the above lines.

The overall mobility of the rats was significantly reduced in the second test in comparison with the first in DAT +/+ rats but not in DAT −/− and DAT +/− rats. Rats with the heterozygous DAT +/− genotype showed the lowest overall mobility in the first test in comparison with both DAT −/− and DAT +/+ genotypes. In this regard, it can be assumed that DAT +/− rats have a higher resistance to external factors (less anxiety), in the case of consuming both the control diet and HFCD (Fig. 3). The consumption of HFCD led to an abolition of the decrease in mobility in DAT +/+ rats. The features in mobility changes in DAT +/− compared to homozygous and wild‐type animals are partially consistent with findings in Adinolfi et al. (2018). With age, the HFCD prevented a decrease in the exploratory and movement activity in the rats of all studied lines.

The level of anxiety of homozygous DAT −/− rats was significantly decreased in comparison with DAT +/+ rats consuming the control diet in the first test (Fig. 2C and D). The consumption of HFCD contributed to a decrease in anxiety with age in DAT +/+ and DAT +/− rats but did not affect DAT −/− rats in this respect. The HFCD also blocked the decline in search and movement activity in the rats of all studied lines with age. Thus, the level of anxiety in the EPM test in DAT +/+ rats was higher than that in DAT +/− and DAT −/− rats. Against the backdrop of HFCD consumption, the DAT +/− rats were less affected compared to DAT −/− rats. The use of HFCD contributed to a decrease in anxiety with age in DAT +/+ and DAT +/− rats but did not affect, in this respect, DAT −/− rats. Similar anxiety reduction and increased resistance to stress in rats of the wild‐type phenotype were also identified in MacKay et al. (2017) against the background of consumption of a high‐calorie diet.

Indicators of the comparative analysis of short‐term memory in the CRPA test suggested that consumption of HFCD together with the decrease in anxiety level shown in the EPM test did not lead to an improvement in short‐term memory in rats with the DAT +/− and DAT −/− genotypes. In the case of DAT +/− heterozygotes in the HFCD group in the second test, the indicators of short‐term memory significantly improved in comparison with the rest of the rats from the HFCD groups.

Therefore, the knockout of the gene for the dopamine transporter (DAT) affects the integral indices (increase in the relative mass of the brain, decrease in the relative mass of the liver and white fat, etc.), the state of neuromotor function, the formation of a short memory trace, and the level of anxiety. Moreover, the consumption of HFCD significantly modifies the intensity and direction of these effects, which may be presumably due to the influence of this diet on the processes of dopamine metabolism in the brain.

In addition, AsAT/AlAT ratio and urea level in blood plasma showed a statistically significant increase in rats of the DAT‐KO knockout line in comparison with DAT +/+ rats regardless of the diet used, which may be the cause of activation of carbohydrate catabolism and alanine in a glucose‐alanine shunt and a source of amino acids in the ornithine cycle in the liver under conditions of genetically mediated elevated energy losses in these animals.

## Conflict of Interest

The authors declare that they have no conflict of interest.
